# Pixel-Level Deep Segmentation: Artificial Intelligence Quantifies Muscle on Computed Tomography for Body Morphometric Analysis

**DOI:** 10.1007/s10278-017-9988-z

**Published:** 2017-06-26

**Authors:** Hyunkwang Lee, Fabian M. Troschel, Shahein Tajmir, Georg Fuchs, Julia Mario, Florian J. Fintelmann, Synho Do

**Affiliations:** 1 Department of Radiology, Massachusetts General Hospital, 25 New Chardon Street, Suite 400B, Boston, MA 02114 USA; 2 Department of Radiology, Charite - Universitaetsmedizin Berlin, Chariteplatz 1, 10117 Berlin, Germany

**Keywords:** Muscle segmentation, Convolutional neural networks, Computer-aided diagnosis (CAD), Computed tomography, Artificial intelligence, Deep learning

## Abstract

Pretreatment risk stratification is key for personalized medicine. While many physicians rely on an “eyeball test” to assess whether patients will tolerate major surgery or chemotherapy, “eyeballing” is inherently subjective and difficult to quantify. The concept of morphometric age derived from cross-sectional imaging has been found to correlate well with outcomes such as length of stay, morbidity, and mortality. However, the determination of the morphometric age is time intensive and requires highly trained experts. In this study, we propose a fully automated deep learning system for the segmentation of skeletal muscle cross-sectional area (CSA) on an axial computed tomography image taken at the third lumbar vertebra. We utilized a fully automated deep segmentation model derived from an extended implementation of a fully convolutional network with weight initialization of an ImageNet pre-trained model, followed by post processing to eliminate intramuscular fat for a more accurate analysis. This experiment was conducted by varying window level (WL), window width (WW), and bit resolutions in order to better understand the effects of the parameters on the model performance. Our best model, fine-tuned on 250 training images and ground truth labels, achieves 0.93 ± 0.02 Dice similarity coefficient (DSC) and 3.68 ± 2.29% difference between predicted and ground truth muscle CSA on 150 held-out test cases. Ultimately, the fully automated segmentation system can be embedded into the clinical environment to accelerate the quantification of muscle and expanded to volume analysis of 3D datasets.

## Introduction

Image segmentation, also known as pixel-level classification, is the process of partitioning all pixels in an image into a finite number of semantically non-overlapping segments. In medical imaging, image segmentation has been considered a fundamental process for various medical applications including disease diagnosis, prognosis, and treatments. In particular, muscle segmentation on computed tomography (CT) for body composition analysis has emerged as a clinically useful risk stratification tool in oncology [1–3], radiation oncology [4], intensive care [5, 6], and surgery [7–10]. Cadaver studies have established muscle cross-sectional area (CSA) at the level of the third lumbar (L3) vertebral body as a surrogate marker for lean body muscle mass [11, 12]. These studies applied semi-automated threshold-based segmentation with pre-defined Hounsfield unit (HU) ranges to separate lean muscle mass from fat. However, segmentation errors require manual correction based on visual analysis by highly skilled radiologists [13]. As a result, semi-automated body composition analysis on large datasets is impractical due to the expense and time required. Thus, there is a role for automated tissue segmentation in order to bring body composition analysis into clinical practice.

Adipose tissue segmentation on CT images is a relatively straightforward process as fat can be thresholded with a consistent HU range [−190 to −30] [14]. Muscle segmentation is less straightforward as muscle and neighboring organs have overlapping HU values [−29 to 150]. Few published strategies exist for automated muscle segmentation with various approaches. A series of publications by Kamiya et al. [15–17] focused on segmentation of a single muscle (psoas major) at L3. Popuri et al. have studied the segmentation of all skeletal muscles visible at the L3 [18] and T4 levels [19, 20]. Their approach involves a deformable shape model based on the ideal muscle appearance with fitting based on a statistical deformation model (SDM). Another study [21] attempted to segment a 3D body CT dataset with seven segmentation classes including fat and muscle by classifying each class using random forest classifiers when given 16 image features extracted from statistical information and filter responses. All these attempts require sophisticated hand-crafted features to define knowledge-based parameters and select constraints for well-formed statistical shape and appearance models. As a result, these approaches cannot be generalized.

Deep learning has demonstrated enormous success in improving diagnostic accuracy, speed of image interpretation, and clinical efficiency for a wide range of medical tasks, ranging from the interstitial pattern detection on chest CT [22] to bone age classification on hand radiographs [23]. Particularly, a data-driven approach with deep neural networks has been actively utilized for several medical image segmentation applications, ranging from segmenting brain tumors on magnetic resonance images [24–26], organs of interest on CT [27, 28], to segmenting the vascular network of the human eye on fundus photography [29]. This success is attributed to its capability to learn representative and hierarchical image features from data [30], rather than relying on manually engineered features based on knowledge from domain experts.

In this study, we propose a fully automated deep segmentation system for the segmentation of muscles on an axial CT slice taken at L3 using the improved fully convolutional network (FCN) [31] and post processing. This system enables real-time segmentation of muscle and possibly fat tissue, facilitating clinical application of body morphological analysis sets.

## Method

### Dataset

#### Data Acquisition and Characteristics

IRB approval was obtained for this retrospective study. Four hundred patients with an abdominal CT and lung cancer treated with either surgery or systemic therapy between 2007 and 2015 were identified in an institutional database. The surgical cohort (tumor stages I, II, and III) represented a cross section of all patients who underwent lung cancer resection at our institution, while the medical cohort were patients who received chemotherapy (tumor stage IV). Only examinations with intravenous contrast were included to ensure consistency of HU values. Four hundred examinations of 200 females and 200 male patients were included in the study, as detailed in Table 1. A test subset of 150 cases was created for evaluating the algorithm performance by taking 25 cases from each BMI category per gender, as explained in “Data Categorization.”

**Table 1:** Patient characteristics of the entire cohort (*n* = 400) and the test subset (*n* = 150)

Patient characteristics	*n* = 400 (entire cohort)	*n* = 150 (test subset)	*p* values
Age, mean (SD) (years)	63 (12)	62 (11)	0.31
Gender, no. (%)			1
Female	200 (50)	75 (50)	
Male	200 (50)	75 (50)	
Height, mean (SD) (cm)	168 (10)	168 (10)	0.70
Weight, mean (SD) (kg)	77 (18)	79 (19)	0.16
Lung cancer treatment, no. (%)			0.78
Systemic therapy	227 (57)	86 (57)	
Surgery	173 (43)	64 (43)	
Lung cancer stage, no. (%)			0.84
I	102 (26)	38 (25)	
II	33 (8)	10 (7)	
III	38 (10)	16 (11)	
IV	227 (57)	86 (57)	

Note that there is no statistically significant difference between the entire cohort and the test subset

Images were acquired for routine clinical care as detailed in Table 2. Scanners were calibrated daily using manufacturer-supplied phantoms to ensure consistency in attenuation measurements in accordance with manufacturer specifications. Full resolution 512 × 512 pixel diagnostic quality CT examinations were loaded onto a research workstation running OsiriX without downsampling (Pixmeo, Bernex, Switzerland). Segmentation maps of skeletal muscle CSA at the level of L3 were created on a single axial image using semi-automated threshold-based segmentation (thresholds −29 to +150 HU). Analyzed muscles included the transversus abdominis, external and internal abdominal obliques, rectus abdominis, erector spinae, psoas major and minor, and quadratus lumborum. A research assistant (initials [JM]) blinded to all other data created the segmentation maps. *All cases were reviewed and corrected as necessary by a fellowship-trained board-certified radiologist* (*initials* [*FJF*] *with 8-years of experience*). A subset of the images were randomly selected and then re-analyzed by a second research assistant (initials [GF]) with an inter-analyst agreement of 0.998. These muscle segmentation maps were used for ground truth labeling during training, testing, and verification.

**Table 2:** Image acquisition parameters

Imaging system	*n* = 400 (entire cohort)	*n* = 150 (test subset)
Tube current, mA (SD)	360.78 (124.10)	363.41 (126.85)
kV, (SD)	120.85 (5.68)	120.67 (5.85)
Oral contrast, no. (%)	191 (48)	70 (47)
Manufacturer, no. (%)		
Siemens	141 (35)	92 (35)
GE	241 (60)	52 (61)
Philips	17 (4)	6 (4)
Toshiba	1 (0)	0 (0)

#### Data Preparation

**Fig. 1:**
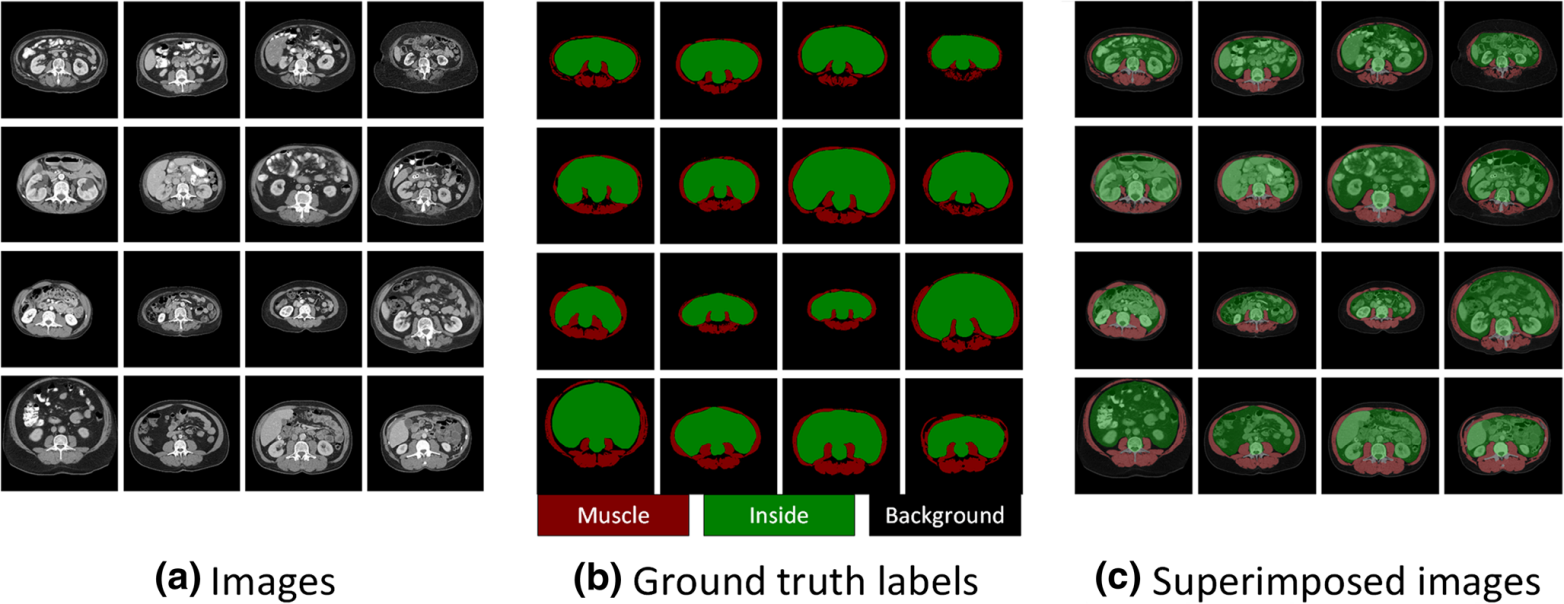
Examples of (**a**) axial images and (**b**) ground truth labels used for training and testing the segmentation convolutional neural network (CNN). (**c**) Superimposed images demonstrate the target output by the CNN. Note that “*Inside*” corresponds to the entire region surrounded by muscle, including organs, fat, and vertebra

We reformatted the manually tuned muscle segmentation maps created by domain experts as described previously into acceptable input for convolutional neural networks (CNN). As shown in Fig. 1, the axial images and their corresponding color-coded images served as original input data and ground truth labels, respectively. The main challenge for muscle segmentation is the accurate differentiation of muscle tissue from neighboring organs due to their overlapping HU ranges. We manually drew a boundary between organs and muscle, setting the inside region as additional segmentation class (“Inside”) in an effort to train the neural network to learn distinguishing features of muscle for a precise segmentation from adjacent organs. The color-coded label images were assigned to pre-defined label indices, including 0 (black) for “Background”, 1 (red) for “Muscle”, and 2 (green) for “Inside”, before passing through CNNs for training as presented in Fig. 1.

#### Data Categorization

**Fig. 2:**
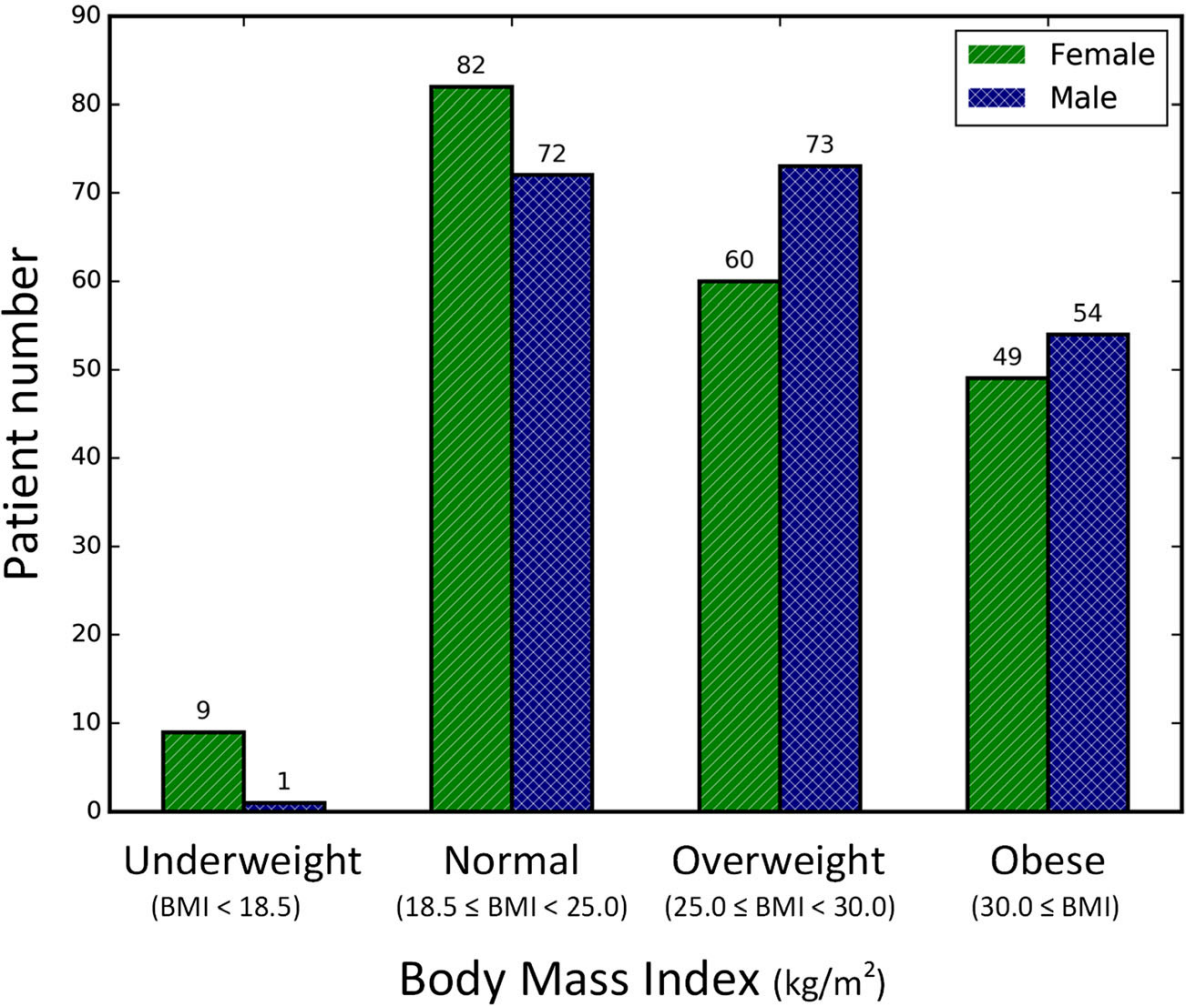
Patients stratification based on gender and body mass index (BMI). For each gender, 25 cases were randomly selected from normal, overweight, and obese weight categories for the testing cohort. Underweight cases were excluded. One hundred fifty total cases were withheld for algorithm testing. The remaining cases were used to train the segmentation convolutional neural network

We hypothesized that differences in body habitus could represent a confounding feature if the network was to be presented unbalanced examples, particularly because prior work has demonstrated that obese patients have higher image noise [32]. To minimize this possibility, the patients were categorized into eight groups based on gender and body mass index (BMI) (Fig. 2). We randomly selected 25 male and 25 female patients from the groups with normal weight, overweight, and obese in order to create a subset of 150 cases to be withheld for testing. All underweight cases were included in the training dataset without being used for testing due to their small number. The other 250 cases were used for training. We chose the best model out of several trained models by selecting the last model after the loss became converged for a sufficiently long period of training time, approximately 500 epochs. The best CNN was evaluated using the held-out test datasets to determine how much the predicted muscle regions overlap with the ground truth. In order to make a fair comparison, we used the same seed value for the random selection from the test dataset for each experiment.

### System Architecture

**Fig. 3:**
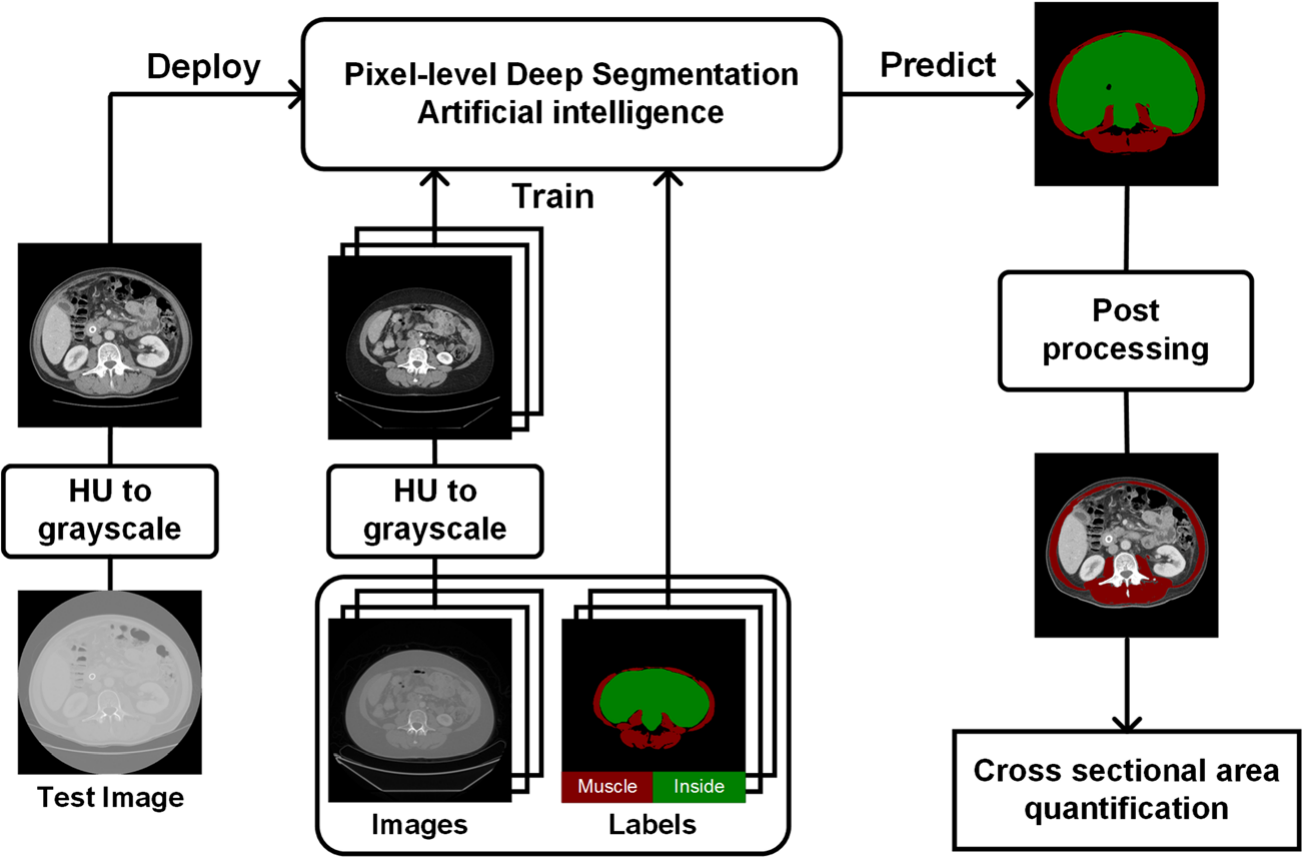
Overview of proposed fully automated deep segmentation system for muscle tissue segmentation. *HU* Hounsfield units

Our proposed fully automated deep segmentation system for muscle segmentation includes grayscale image conversion using the best combination of window settings and bit depth per pixel with post processing to correct erroneous segmentation (Fig. 3).

#### Segmentation AI: Fully Convolutional Network

**Fig. 4:**
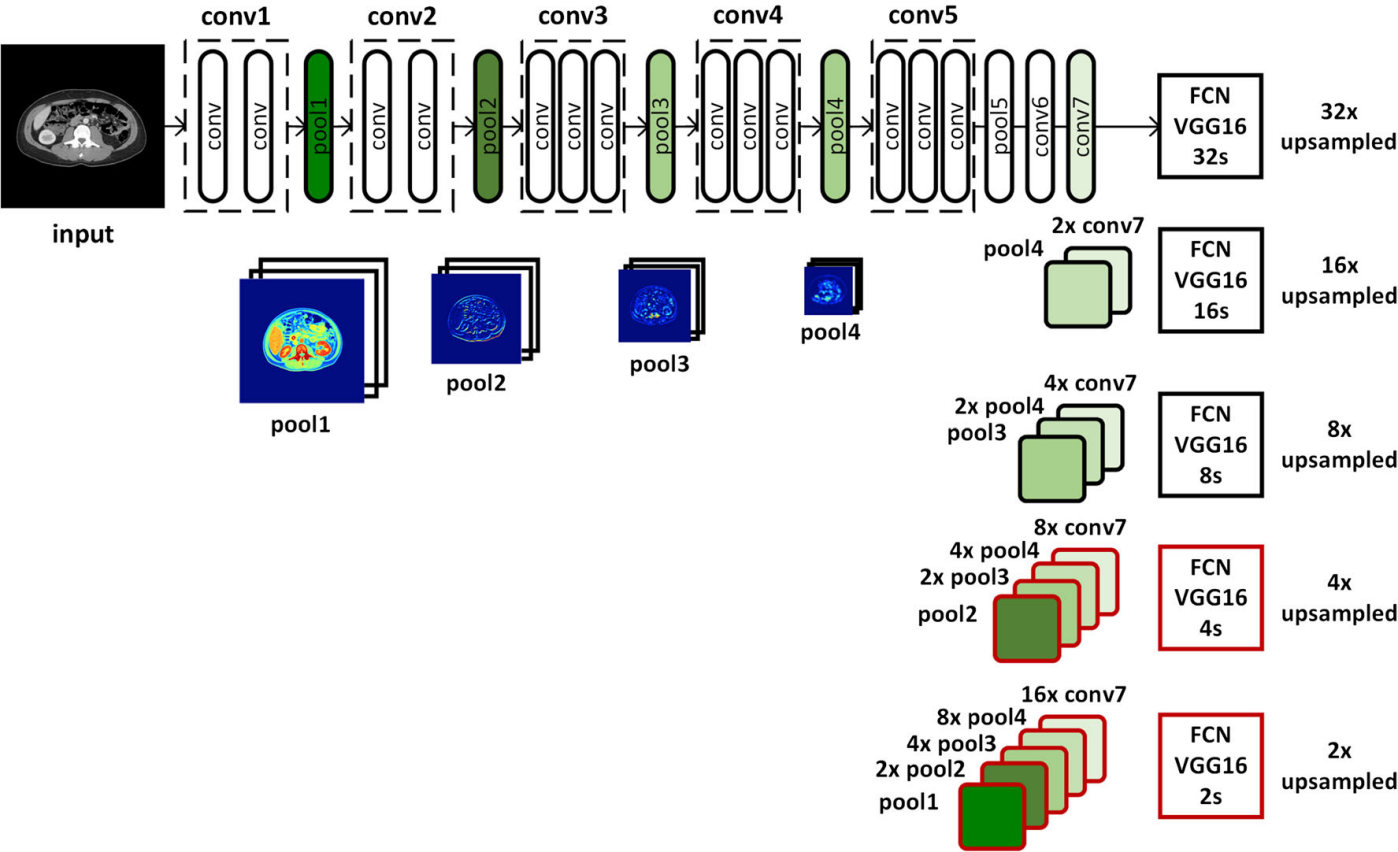
Overview of the proposed fully convolutional network (FCN). FCN-32s, FCN-16s, and FCN-8s appeared in the original FCN implementation [31]. The *red-rimmed FCN-4s* and *FCN-2s* are our extended version of FCN required for more detailed and precise muscle segmentation

Several state-of-the-art deep learning algorithms have been validated for natural image segmentation applications [31]. We chose to develop our muscle segmentation model based on a fully convolutional network (FCN) for three reasons: First, a set of convolutional structures enables learning highly representative and hierarchical abstractions from whole-image input without excessive use of trainable parameters thanks to the usage of shared weights. Second, fine-tuning the trainable parameters of the FCN after weights that are initialized with a pre-trained model from a large-scale dataset allows the network to find the global optimum with a fast convergence of cost function when given a small training dataset. Third, the FCN intentionally fuses different levels of layers by combining coarse semantic information and fine appearance information to maximize hierarchical features learned from earlier and later layers. As shown in Fig. 4, FCN-32s, FCN-16s, and FCN-8s fuse coarse-grained and fine-grained features and upsample them at strides 32, 16, and 8, for further precision. Prior implementations of FCN describe further fusion of earlier layers beyond pool3; however, this was not pursued in their implementation due to only minor performance gains [31]. However, we decided to extend to FCN-4s and FCN-2s (highlighted in red in Fig. 4) by fusing earlier layers further because muscle segmentation requires finer precision than stride 8.

#### Image Conversion: HU to Grayscale

**Fig. 5:**
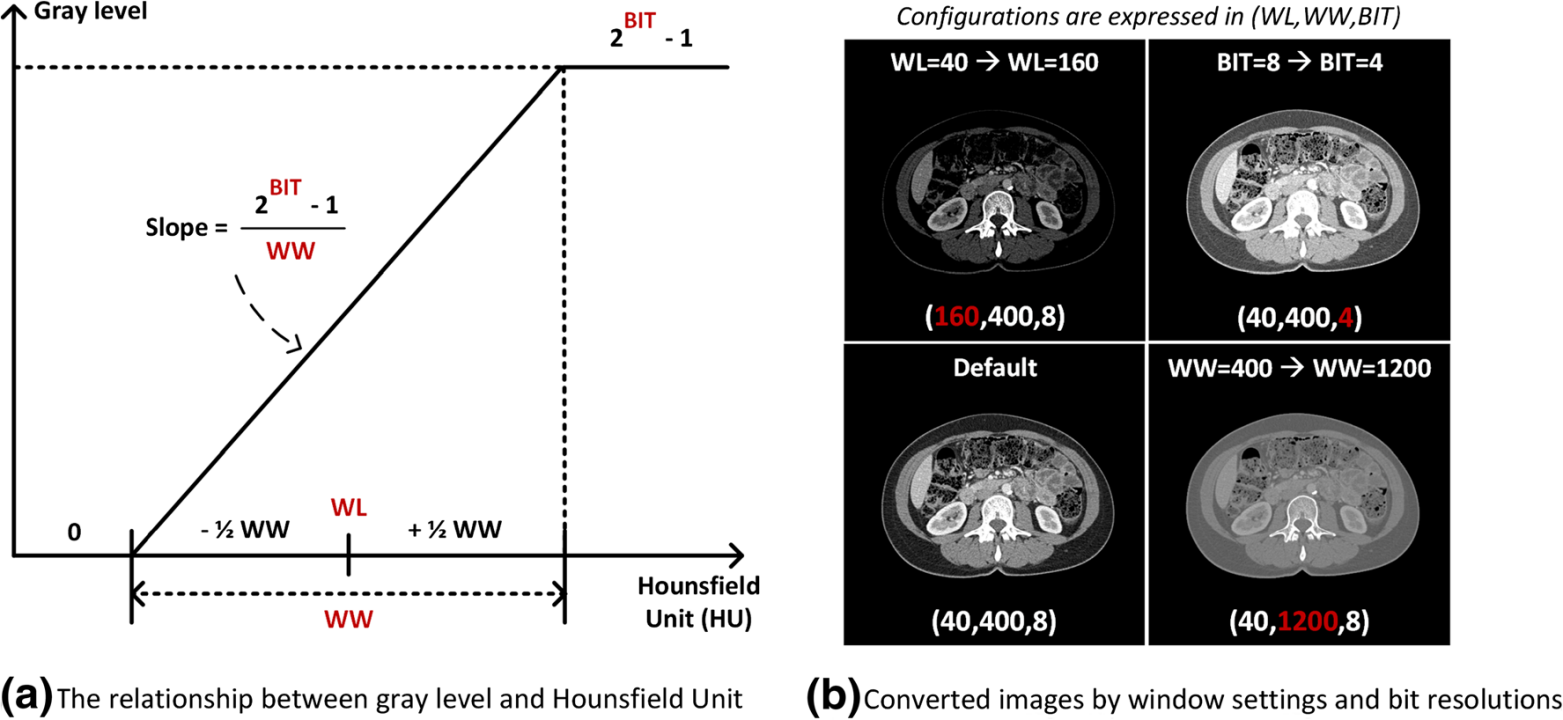
(**a**) The relationship between gray level and Hounsfield units (HU) determined by window level (WL), window width (WW), and bit depth per pixel (BIT). (**b**) The effect of different WL, WW, and BIT configurations on the same image

Medical images contain 12 to 16 bits per pixel, ranging from 4096 to 65,536 shades of gray per pixel. A digital CT image has a dynamic range of 4096 gray levels per pixel (12 bits per pixel), far beyond the limits of human perception. The human observer can distinguish many hundred shades of gray, and possibly as high as 700–900, but substantially less than the 4096 gray levels in a digital CT image [33]. Displays used for diagnostic CT interpretation support at most 8 bits per pixel, corresponding to 256 gray levels per pixel. To compensate for these inherent physiologic and technical limitations, images displayed on computer monitors can be adjusted by changing the window level (WL) and window width (WW), followed by assigning values outside the window range to minimum (0) or maximum (2^BIT^-1) value, as described in Fig. 5a. The WL—the center of the window range—determines which HU values are converted into gray levels. The WW determines how many of HU values are assigned to each gray level, related to the slope of the linear transformation shown in Fig. 5a. BIT, the available number of bits per pixel, determines how many shades of gray are available per pixel. The effects of the three configurations on image appearance are demonstrated with four examples images in Fig. 5b. The optimal window setting configuration is dependent on the HUs of the region of interest (ROI) and the intrinsic image contrast and brightness. These settings are ultimately workarounds for the constraints of human perception. However, computer vision does not necessarily have these limitations.

Most prior investigations have converted CT images to grayscale with the commonly used HU range for the target tissue or organ without studying the effect of window settings on the performance of their algorithms. While recent work has identified that image quality distortions limit the performance of neural networks [34] in computer vision systems, the effect of window setting and bit resolution on image quality is often overlooked in medical imaging machine learning. Therefore, we evaluated the effects of window and BIT settings on segmentation performance by sweeping different combinations of window configurations and bit depth per pixel.

#### Comparison Measures

The primary comparison measure utilizes the Dice similarity coefficient (DSC) to compare the degree of overlap between the ground truth segmentation mask and the FCN-derived mask, calculated as Eq. 1.

1 $$ \mathrm{DSC}=\frac{2\times \left|\mathrm{ground}\ \mathrm{truth}\cap \mathrm{predict}\right|}{\left|\mathrm{groiund}\ \mathrm{truth}\right|+\left|\mathrm{predict}\right|} $$

An additional comparison measure was the cross-sectional area (CSA) error, calculated as Eq. 2. This represents a standardized measure of the percentage difference in area between the ground truth segmentation mask and the FCN-derived mask.

2 $$ \mathrm{CSA}\ \mathrm{error}\ \left(\%\right)=\frac{\left|\mathrm{ground}\ \mathrm{truth}-\mathrm{predict}\right|}{\mathrm{ground}\ \mathrm{truth}}\times 100 $$

#### Intramuscular Fat Post Processing

Muscle tissue HUs do not overlap with adipose tissue HUs. As a result, a binary image of fat regions extracted using HU thresholding can be utilized to remove intramuscular fat incorrectly segmented as muscle.

#### Validation and Quality Control

Subsequent to post processing, the results of the test subset were visually analyzed by a research assistant together with a fellowship-trained board-certified radiologist (initials [FJF], 8 years of experience). Common errors were identified and occurrence was noted for each image.

#### Training

We trained the models by a stochastic gradient descent (SGD) with a momentum of 0.9 and with a minibatch size of 8 to achieve full GPU utilization. As performed in [31, 35], we utilized a fixed, tiny learning rate and weight decay because training is highly sensitive to hyperparameters when unnormalized softmax loss is used. We empirically found that a learning rate of 10^−10^ and a weight decay of 10^−12^ were optimal for our application to obtain stable training convergence at the cost of convergence speed. Since training losses eventually converged if the models were trained for sufficient period of epochs, all models in this paper were trained for 500 epochs and the last model was selected without a validation phase to evaluate performance on our held-out test subset. All experiments were run on a Devbox (NVIDIA Corp, Santa Clara, CA) containing four TITAN X GPUs with 12GB of memory per GPU [36] and using Nvidia-Caffe (version 0.15.14) and Nvidia DIGITS (version 5.1).

#### Statistical Analysis

Descriptive data were presented as percentages for categorical variables and as means with standard deviation (SD) for continuous variables. We used two-tailed statistical tests with the alpha level set at 0.05. We performed Student’s *t* test for normally distributed values. Dichotomous variables were compared using the Mann Whitney *U* test and ordinal variables were compared using the Kruskal Wallis test. Inter-analyst agreement was quantified with intraclass correlation coefficients (ICC). All statistical analyses were performed using STATA software (version 13.0, StataCorp, College Station, TX).

### Experiments

#### Fully Convolutional Network

To identify the best performing fully convolutional network, five models of increasing granularity—FCN-32s, FCN-16s, FCN-8s, FCN-4s, and FCN-2s—were trained and evaluated using the test dataset at 40,400 and 8 bits per pixel by measuring the DSC and CSA error between ground truth and predicted muscle segmentation. These results were compared to the HU thresholding method, selecting HU ranging from −29 to 150 to represent lean muscle CSA.

#### Image Conversion: HU to Grayscale

Subsequently, we compared the performance of the best FCN model (FCN-2s) with seven different combinations of window settings for each bit depth per pixel—(40,400), (40, 240), (40,800), (40,1200), (−40,400), (100,400), and (160,400) expressed in WL and WW and 8, 6, and 4 bit resolutions per pixel. The selected window ranges cover the HU range of lean tissue [−29 to 150] for a fair comparison to see if partial image information loss degrades model performance. These window settings contain extreme window ranges as well as typical ones. For example, the window setting (40,240) has a range of −80 to 160 HU values, which corresponds to almost the HU range of lean muscle, while the configuration (40,1200) converts all HU values between −560 and 1240 into shades of gray resulting in low image contrast.

## Results

### FCN Segmentation Performance

**Fig. 6:**
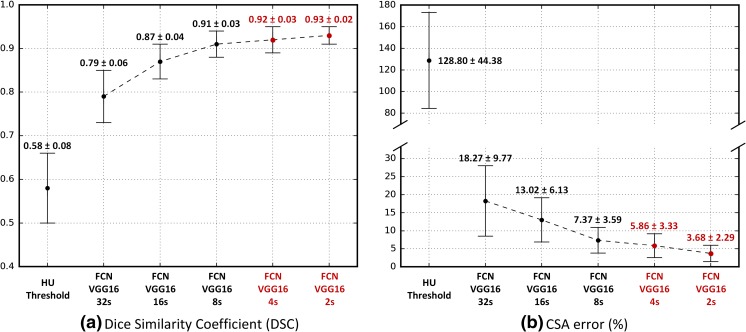
Comparison of the HU thresholding method and five different FCNs. (**a**) Dice similarity coefficient (DSC) and (**b**) cross-sectional area (CSA) error between ground truth manual and predicted muscle segmentation areas. All numbers are reported as mean ± SD

**Fig. 7:**
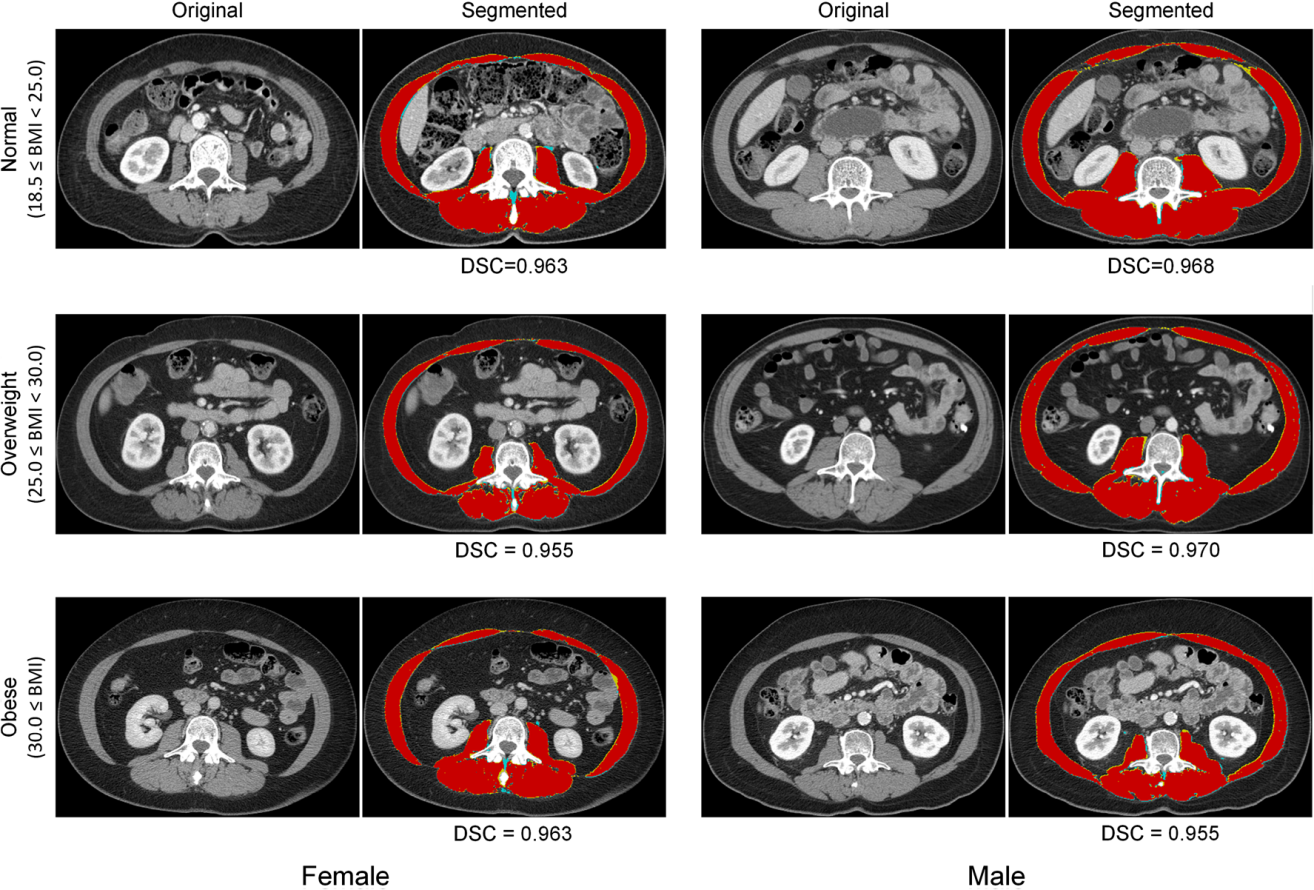
Six examples of the better segmented CT images for six groups according to gender and BMI. Dice similarity coefficient (DSC) is marked on each segmented image *above*. Oversampled regions are colored in *blue*, undersampled areas are colored in *yellow*, and correctly segmented areas are colored in *red*

The five different FCN models were compared to the previously described HU thresholding method. Performance was evaluated using the DSC and muscle CSA error and detailed in Fig. 6. Even the most coarse-grained FCN model (FCN-32s) achieved 0.79 ± 0.06 of DSC and 18.27 ± 9.77% of CSA error, markedly better than the HU thresholding method without human tuning. Performance increased as the number of features of different layers was fused. The most fine-grained FCN model achieved DSC of 0.93 and CSA error of 3.68% on average, representing a 59% improvement in DSC and an 80% decrease in CSA error when compared to the most coarse-grained model. The representative examples are detailed in Fig. 7 to visually show the performance of FCN-2s segmentation.

### Effect of Window and Bit Settings on Segmentation Performance

**Fig. 8:**
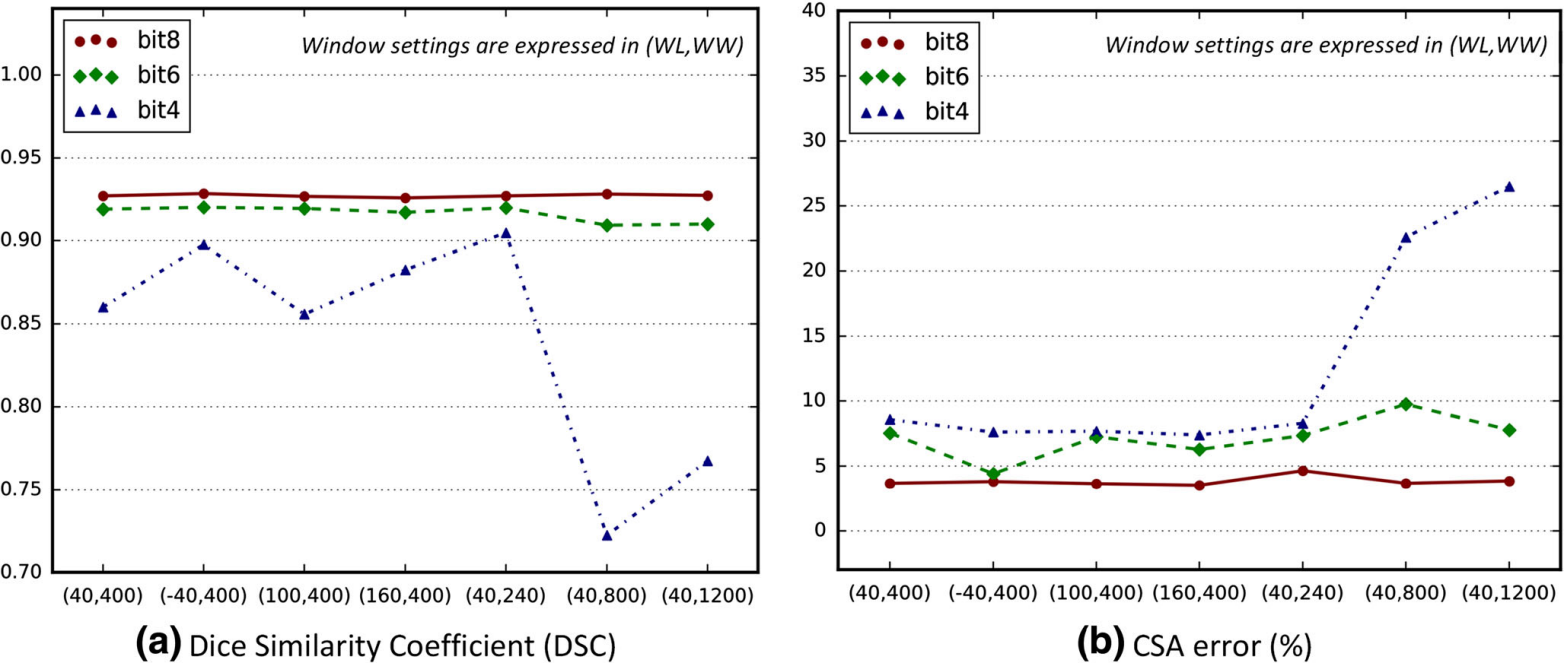
Performance of FCN-2s when input images are generated with different window settings (WL, WW) for each bit depth per pixel (BIT). The selected window settings were (40,400), (−40,400), (100,400), (160,600), (40,240), (40,800), and (40,1200)

Results of the systematic experiment comparing seven different combinations of window settings for each bit depth per pixel are presented in Fig. 8. The DSC and CSA error were not meaningfully influenced by changes in window ranges as long as 256 gray levels per pixel (bit8) were available. When 6-bit depth per pixel was used, performance was similar compared to the results of 8-bit cases. However, model performance deteriorated when 8-bit pixels were compressed to 4-bit pixels.

### Deployment Time

Segmentation was performed using a single TITAN X GPU. Segmentation took 25 s on average for 150 test images, corresponding to only 0.17 s per image.

### Statistical Analysis of Model Segmentation Errors

**Fig. 9:**
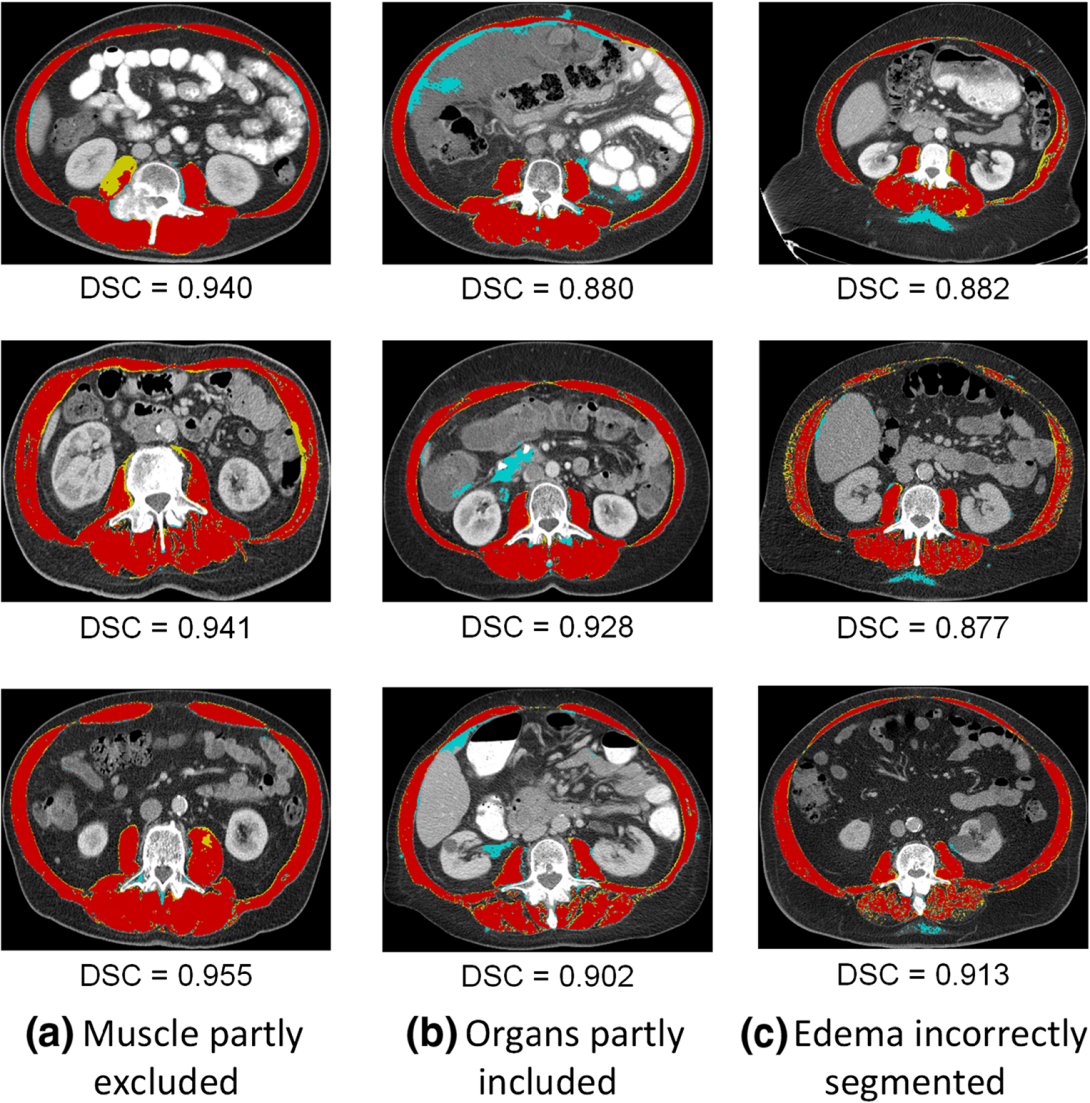
Segmentation errors most commonly presented as muscle partly excluded (**a**), organs partly included (**b**), and edema mischaracterized as muscle (**c**). Oversampled regions are colored in *blue*, undersampled areas are colored in *yellow*, and correctly segmented areas are colored in *red*

In the majority of cases (*n* = 128), FCN CSA was smaller than ground truth CSA, while only few cases resulted in oversegmentation (*n* = 22; *p* < 0.0001). Review of incorrectly segmented images identified three main errors: incomplete muscle segmentation (*n* = 58; 39% of test cases), incorrect organ segmentation (*n* = 52; 35%), and subcutaneous edema mischaracterized as muscle (*n* = 17; 11% of test cases). Representative examples of these errors are demonstrated in Fig. 9.

### Obesity

To evaluate the influence of obesity on the performance of the segmentation algorithm, segmentation results of patients with BMI >30 kg/m^2^ were compared to those of patients with BMI <30 kg/m^2^. The average DSC was 0.93 in non-obese patients, but only 0.92 in obese patients, a statistically significant difference (*p* = 0.0008). The incorrect inclusion of subcutaneous soft tissue edema into muscle CSA was more common in obese patients than in non-obese patients (*p* = 0.018). However, inclusion of adjacent organs into muscle CSA (*p* = 0.553) and incomplete muscle segmentation (*p* = 0.115) were not significantly associated with obesity. There was no statistically significant association between obesity and CSA error (*p* = 0.16).

### Oral Contrast

Forty-eight percent of the cohort received oral contrast in addition to intravenous contrast. The ratio was the same in the training and testing datasets. There was no statistically significant association between the presence or absence of oral contrast and segmentation performance measured as DSC (*p* = 0.192) or CSA error (*p* = 0.484), probably because the network became invariant to its presence in the balanced cohorts.

## Discussion

We have developed an automated system for performing muscle segmentation at the L3 vertebral body level using a fully convolutional network with post processing at a markedly faster deployment time when compared to conventional semi-automated methods.

Our model was derived from a highly granular fully convolutional network and compared to the semi-automated HU thresholding method which requires tedious and time-consuming tuning of erroneous segmentation by highly trained human experts. When compared to the HU thresholding method without human tuning, even the coarsest FCN had markedly better performance. It is not surprising as HU thresholding is so inaccurate, as it includes overlapping HU ranges between organs and muscle. However, by combining hierarchical features and different layers of increasing granularity, our model was able to extract semantic information, overall muscle shape, fine-grained appearance, and muscle textural appearance. These results persisted even when varying the WL and WW into ranges unsuitable for the human eye. Changes in WW had greater effects on segmentation performance than WL, particularly when the number of gray shades was small (bit6 and bit4). These results imply that this network’s performance depends mostly on image contrast and possibly due to the number of HU values assigned to a single gray level, rather than inherent image brightness. It also implies that preserving image information using the original 12-bit resolution with 4096 shades of gray could provide considerable performance gains by allowing the network to learn other significant identifying features of muscle which are lost in the conversion to 8 bits. These results are consistent with other published findings that CNNs are excellent at textural analysis [37, 38].

### Deployment Time

Accurate segmentation of muscle tissue by the semi-automated HU thresholding method requires roughly 20–30 min per slice on average [18]. Algorithms proposed in most prior works [16, 18, 19] required between 1 and 3 min per slice. More recent works have reported that their algorithms require only 3.4 s [21] and 0.6 s per image on average. To the best of our knowledge, our model is the fastest reported segmentation algorithm for muscle segmentation and needs only 0.17 s per slice on average. Segmenting 150 test images can be performed in 25 s. This ultra-fast deployment can allow real-time segmentation in clinical practice.

### Clinical Applications

Muscle CSA at L3 has been shown to correlate with a wide range of posttreatment outcomes. However, integration of muscle CSA measurements in clinical practice has been limited by the time required to generate this data. By dropping the calculation time from 1800 to 0.17 s, we can drastically speed up research into new applications for morphometric analysis. CT is an essential tool in the modern healthcare arena with approximately 82 million CT examinations performed in the USA in 2016 [39]. In lung cancer in particular, the current clinical paradigm has been on lesion detection and disease staging with an eye toward treatment selection. However, accumulating evidence suggests that CT body composition data could provide objective biological markers to help lay the foundation for the future of personalized medicine. Aside from preoperative risk stratification for surgeons, recent work has used morphometric data to predict death in radiation oncology and medical oncology [4]. Our system has the great advantage of not requiring a special protocol (other than intravenous contrast) and could derive muscle CSA from routine CT examinations near-instantaneously. This would enable body composition analysis of the vast majority of CT examinations.

### Limitations

While the system has great potential for accelerating calculation of muscle CSA, there are important limitations. The network statistically tends to underestimate muscle CSA. This is probably due to a combination of overlapping HUs between muscle and adjacent organs and variable organ textural appearance. On the other end of the spectrum, segmentation is also confused by the radiographic appearance of edema particularly in obese patients, which has a similar HU range to muscle, leading to higher CSA than expected. Extensive edema tends to occur in critically ill patients, leading to potentially falsely elevated CSA in patients actually at higher risk for all interventions.

The average age of our cohort is 63 years. While this is representative of the lung cancer population, it may limit the generalizability of our system for patients with different diseases and age groups. Further training with data from a wider group of patients could enable the algorithms to account for these differences. In addition, the network should be trained to segment CT examinations performed without intravenous contrast and ultra-low radiation dose.

### Future Directions

*The muscle segmentation AI can be enhanced further by using the original 12-bit image resolution with 4096 gray levels which could enable the network to learn other significant determinants which could be missed in the lower resolution*. In addition, an exciting target would be adipose tissue segmentation. Adipose tissue segmentation is relatively straightforward since fat can be thresholded within a unique HU range [−190 to −30]. Prior studies proposed creating an outer muscle boundary to segment HU thresholded adipose tissue into visceral adipose tissue (VAT) and subcutaneous adipose tissue (SAT). However, precise boundary generation is dependent on accurate muscle segmentation. By combining our muscle segmentation network with a subsequent adipose tissue thresholding system, we could quickly and accurately provide VAT and SAT values in addition to muscle CSA. Visceral adipose tissue has been implicated in cardiovascular outcomes and metabolic syndrome, and accurate fat segmentation would increase the utility of our system beyond cancer prognostication [40]. Ultimately, our system should be extended to whole-body volumetric analysis rather than axial CSA, providing rapid and accurate characterization of body morphometric parameters.

## Conclusion

We have created an automated, deep learning system to automatically detect and segment the muscle CSA of CT slices at the L3 vertebral body level. This system achieves excellent overlap with hand-segmented images with an average of less than 3.68% error while rapidly accelerating segmentation time from 30 min to 0.17 s. The fully automated segmentation system can be embedded into the clinical environment to accelerate the quantification of muscle to provide advanced morphometric data on existing CT volumes and possible expanded to volume analysis of 3D datasets.
